# Lipopolysaccharide-Binding Protein for Monitoring of Postoperative Sepsis: Complemental to C-Reactive Protein or Redundant?

**DOI:** 10.1371/journal.pone.0023615

**Published:** 2011-08-25

**Authors:** Klaus Tschaikowsky, Monika Hedwig-Geissing, Joachim Schmidt, Giovanni G. Braun

**Affiliations:** Department of Anesthesiology, University of Erlangen-Nürnberg, Erlangen, Germany; Montana State University, United States of America

## Abstract

**Introduction:**

To prospectively evaluate the performance of Lipopolysaccharide-Binding Protein (LBP) in prediction of hospital mortality and its correlation to C-reactive Protein (CRP), we studied sixty consecutive, postoperative patients with sepsis admitted to the university hospital intensive care unit.

**Measurements and Methods:**

Plasma LBP and CRP were serially measured from day(d)1 (onset of sepsis) to d14 in parallel with clinical data until d28. Predictive value and correlation of LBP and CRP were analyzed by Receiver Operating Characteristic (ROC) curve analysis and Pearson's test, respectively.

**Main Results:**

LBP and CRP showed the highest levels on d2 or d3 after the onset of sepsis with no significant difference between survivors and nonsurvivors. Only at d7, nonsurvivors had significantly (p = .03) higher levels of CRP than survivors. Accordingly, in ROC analysis, concentration of CRP and LBP on d7 poorly discriminated survivors from nonsurvivors (area under curve = .62 and .55, respectively) without significant difference between LBP- and CRP-ROC curves for paired comparison. LBP and CRP plasma levels allocated to quartiles correlated well with each other (r^2^ = .95; p = .02). Likewise, changes in plasma concentrations of LBP and CRP from one observation to the next showed a marked concordance as both parameters concomitantly increased or decreased in 76% of all cases.

**Conclusions:**

During the first 14 days of postoperative sepsis, LBP plasma concentrations showed a time course that was very similar to CRP with a high concordance in the pattern of day-to-day changes. Furthermore, like CRP, LBP does not provide a reliable clue for outcome in this setting.

## Introduction

Sepsis is still the main cause of death in surgical intensive care units with a continuously increasing incidence and a mortality rate ranging from 30% to 60% depending on sepsis severity and the days of hospital stay [1], [2]. Therefore, both an early diagnosis and prognosis of sepsis are of utmost importance to control efficacy of antibiotic and surgical therapy, to manage further diagnostics and interventions and to optimize cost containment. To identify patients that are at high risk to succumb to sepsis, however, is difficult, due to a vast variety of influencing factors (e.g. age, underlying disease, co-morbidity, focus and type of infection, readiness and adequacy of therapy). Clinical and routine labarotory signs, like fever and leukocytosis, respectively, as well as clinical scores like APACHE II and SAPS II are not always helpful for outcome prediction [3], [4]. Therefore biomarkers that are released during the inflammatory response, like PCT, IL6 and CRP, have been investigated and suggested as useful parameters to determine the outcome of septic patients [4], [5], [6], [7], [8], [9], [10], [11].

However, there is no generally accepted marker for monitoring the evolution of sepsis. More recently, Lipopolysaccharide-binding protein (LBP) has been proposed as a sensitive marker for bacterial infection [12], [13] and possibly useful follow-up parameter in detection and resolution of sepsis [14], [15]. Like CRP, LBP is an acute phase protein, that is produced by hepatocytes as well as epithelial cells of the intestine and the lungs [16], [17] after induction by interleukin-(IL)-6 and IL-1, and a key participant in the innate immune response to Gram-negative, Gram-positive bacterial and fungal infections. Due to a binding site for the lipid A moiety of Lipopolysaccharide (LPS) from Gram-negative bacteria [18], LBP facilitates the transfer of LPS to the membrane-bound CD14-receptor [19]. Thereby low concentrations of LBP enhance LPS-induced cell activation and may induce inflammation at local sites of infection, whereas higher concentrations of LBP can neutralize LPS-induced activation and may prevent systemic inflammation [20]. LBP is constitutively present in human plasma at low concentrations (3–15 µg/ml) [21] and can increase up to 200 µg/ml during the acute phase response [22], [23]. There are several studies investigating the role of LBP in outcome prediction in critically ill patients with sepsis and infection, however, in part with inconsistent result [15], [22], [23], [24]. C-reactive protein (CRP) is a well established parameter to detect local infection and has also been used for many years in monitoring the inflammatory response to sepsis [15], [22], [24].

In the present study we compared the time course of LBP and CRP plasma levels in survivors and nonsurvivors during the first 14 days of postoperative sepsis and examined the performance of both markers regarding outcome prediction. Moreover, we studied whether LBP can provide useful information in addition to CRP and determined the correlation of LBP and CRP and their concordance concerning day-to-day changes of plasma concentrations.

## Materials and Methods

### Objectives

We conducted a prospective, observational study at the University Hospital of Erlangen-Nuernberg, Germany, a 1400-bed tertiary care hospital involving patients with sepsis, severe sepsis and septic shock. Surgical adult patients postoperatively admitted to the interdisciplinary operative ICU (24 bed) after elective major abdominal or thoracic surgery, were included as soon as they met the criteria of sepsis, as defined by the International Sepsis Definitions Conference [25]. Patients were observed for 28 days from enrollment, or until death or discharge from the hospital if either occurred before day 28. They were classified as survivors and nonsurvivors of sepsis according to the outcome at day 28 after study enrollment. The primary study endpoint was 28-day mortality from sepsis.

### Ethics

The study was approved by the Institutional Ethics Committee (Ethik-Kommission der Medizinischen Fakultät der Universität Erlangen-Nürnberg) according to the International Declarations of Helsinki and Tokyo (approval No.: 3298). For participation in the study, informed written consent was obtained from all patients, legal representatives or next of kin.

### Study protocol

At time of enrollment, all included patients had to have a microbiologically confirmed or definite clinical evidence of infection and at least two of the following criteria of a Systemic Inflammatory Response Syndrome (SIRS) within a few hours, not exceeding 24 hours: core body temperature >38.0°C or <35.6°C; tachycardia >90 bpm; tachypnea >20 breaths/min or need for mechanical ventilation; leukopenia (WBC<4000/µl) or leukocytosis (WBC>10000/µl) or more than 10% unsegmented neutrophils. At the onset of sepsis, the severity of the patient's condition was determined by using Acute Physiology and Chronic Health Evaluation II (APACHE II) score. Patients who died of causes clearly not related to sepsis were excluded from outcome analysis (dropouts). All patients were daily screened for the presence of clinical and analytical signs of sepsis and, when indicated, blood cultures, swabs, aspiration or biopsies of suspected sites of infection were obtained to ensure early identification of causative microorganisms. Broad spectrum antibiotic therapy was administered to all patients as soon as sepsis was suspected and adapted according to the antibiogram as soon it was obtained. Diagnostic procedures, e.g. blood gases, laboratory and imaging exams, and supportive therapy (noradrenalin, dobutamine) were performed as clinically indicated. None of the patient studied received Activated Protein C, whereas all patients with septic shock were treated with low dose corticosteroids as adjunctive therapy.

### Measurements and data collection

Within 12 h after study entry, serial, heparinized blood samples were drawn (Heparin-Monovette, Sarstedt, Nuernbrecht, Germany) via an arterial line for inflammation marker measurements on day 1 (i.e. onset of severe sepsis), and at 7–8 a.m. on days 2, 3, 5, 7, 10, and 14. In addition, clinical data were recorded daily during follow-up including demographic data, diagnosis, surgical intervention, site of infection and results from microbiological cultures. Plasma was analyzed the same day or stored at −20°C until further analysis. CRP was measured using a turbidimetric assay with a detection limit of 1 mg/l. Plasma levels of LBP were measured using a semiautomated, chemiluminescent immunoassay (Immulite™, Siemens Healthcare Diagnostics, Eschborn, Germany) with an assay sensitivity of 0.2 µg/ml. Trained laboratory technicians, blinded to the patient's clinical course, treatment assignments and outcome of the patients performed all measurements. Clinicians responsible for the care of the patients were aware of CRP, but unaware of LBP and the data evaluation.

### Statistics

Variables with nominal scale (sex) are described using absolute and relative frequencies. Kolmogorov-Smirnov test was employed to verify the normality of distribution of continuous variables. For univariate description of normally distributed variables, mean values and standard deviation (SD) are given. For univariate description of non-normally distributed variables, median with 25–75 interquartile ranges (boxes) and 5th and 95th percentile (whiskers) were used. To compare concentrations along time within groups Kruskal-Wallis analysis of variance followed by Mann-Whitney U test were applied. Comparisons between survivors and nonsurvivors were performed by using Mann-Whitney U test. To correct for multiple testing, Bonferroni correction was performed. Univariate analysis of predictive accuracy of CRP and LBP plasma concentrations in discriminating survivors from nonsurvivors was done by using receiver operating characteristic (ROC) curves with the area under curve (AUC) as measure of overall performance. The relationship between CRP and LBP plasma concentrations were analyzed by simple linear regression of CRP on LBP after allocating the levels to quartiles (Q1: 0–25th, Q2: 26th–50th, Q3: 51st–75th, Q4: 76th–100th percentile). Correlation between CRP and LBP levels transformed to quartile numbers was investigated by Pearson's test. P-values of 0.05 or less were considered significant. All analyses were done using Statistica (version 6.0, StatSoft, Tulsa, OK, USA) and MedCalc (version 11.1.1, MedCalc Software, Mariakerke, Belgium).

## Results

**Figure 1:**
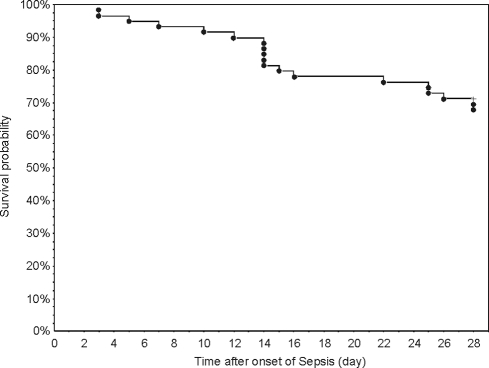
Kaplan-Meier Plots showing the survival rate of the entire study group during 28 days of observation. Before day 14, only 6 (10%) out of 59 patients died. In the third and forth week after onset of sepsis, 7 (12%) and 6 (10%) patients died, respectively. Forty (68%) out of 59 patients survived day 28.

Sixty consecutive patients meeting the criteria of sepsis, severe sepsis or septic shock after abdominal or thoracic surgery and admitted to the interdisciplinary ICU were screened for eligibility. One patient was excluded from analysis due to death not related to sepsis (pulmonary embolism). The demographics and clinical characteristics of the fifty-nine patients that were included in the per-protocol analysis are shown in Table 1. There were 40 (68%) survivors (group S) and 19 (32%) nonsurvivors of sepsis (group NS) 28 days after onset of sepsis (day 1) (Figure 1). The two groups had similar demographic and clinical characteristics including site of infection, surgical intervention and identified microorganisms. Both in S and NS, Gram-negative bacteria were identified in less than 50% of the patients. Likewise, severity of illness at onset of sepsis, as judged by the APACHE II score, was similar both in sepsis survivors and nonsurvivors. For the entire population studied, the APACHE II score at day 1 was 22.5±6.3 (mean ± SD) due to a high percentage (95%) of patients with severe sepsis and septic shock on admission. Within 3 days after admission, all patients enrolled were intubated, mechanically ventilated and received catecholamines (dobutamine, noradrenalin) to keep the sytemic blood pressure above 90 mmHg., and thus fulfilled the criteria of septic shock. All deaths were due to multiple organ failure.

**Table 1:** Demographic and (post)operative characteristics of patients enrolled in the study.

	Survivors	Nonsurvivors	
	(n = 40) 68%	(n = 19) 32%	
Age (yrs)	64.1±16.2	61.5±18.5	p>0.5
Weight (kg)	78.4±2.4	80.6±2.1	p>0.5
Height (cm)	173.3±9.4	171.1±9.1	p>0.5
Gender (%) male	75	74	
APACHE II score (day1)	20.5±6.6	23.5±5.9	p = .098
**Cause of death**			
MODS	n.a.	19	
**Surgery**			
Esophagus	8 (20%)	3 (16%)	
Gastrointestinal	17 (43%)	9 (47%)	
Liver/Gall	4 (10%)	2 (11%)	
Pancreas	4 (10%)	1 (5%)	
Other	7 (18%)	4 (21%)	
**Site of Infection**			
Lung only	7 (18%)	4 (21%)	
Abdomen only	10 (25%)	5 (26%)	
Combined	19 (48%)	8 (42%)	
Other	4 (10%)	2 (11%)	
**Identified Organisms**			
Bacteria Gram positive only	11 (28%)	5 (26%)	
Bacteria Gram negative only	15 (38%)	8 (42%)	
Polymicrobial	10 (25%)	5 (26%)	
Other (unidentified, fungi only)	4 (10%)	1 (5%)	

Values are mean ± SD or actual numbers (percentages) of postoperative patients with sepsis (per protocol analysis, n = 59), assigned to survivors (n = 40) or nonsurvivors (n = 19) according to their survival on day 28 after onset of sepsis. Indicated surgery refers to the main organ operated prior to study enrollment. n.a. not applicable. There were no significant differences between the groups.

### Time course of LBP and CRP plasma concentrations during the first 14 days of sepsis

**Figure 2:**
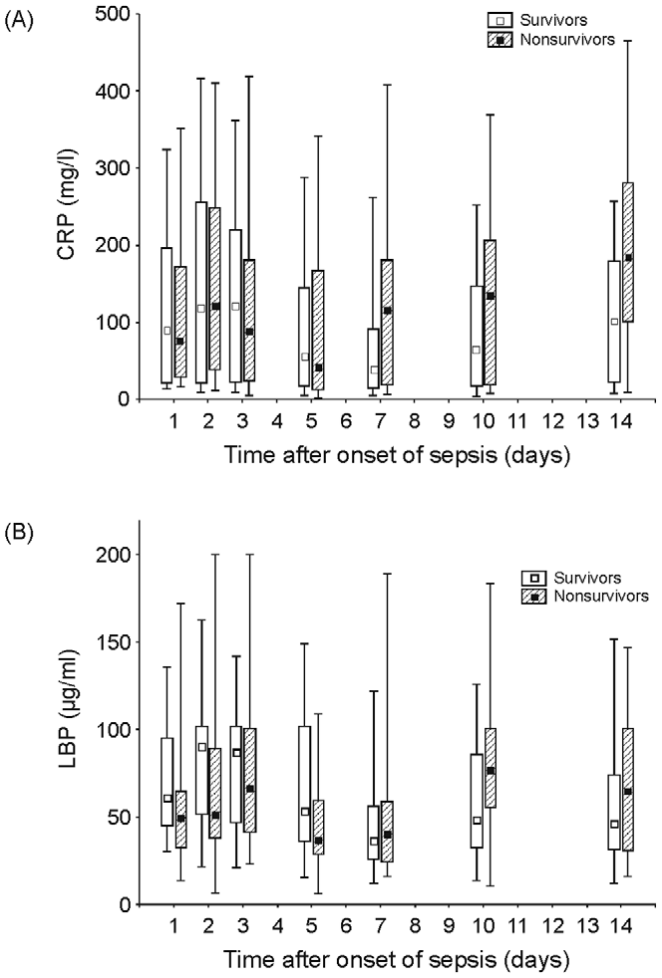
**A.** Time course of CRP plasma concentrations in postoperative septic patients during the first 14 days of sepsis (n = 59). Data are presented as box-plots (open, survivors, n = 40; hatched, nonsurvivors, n = 19) depicting the lower and upper quartiles (boxes) and the 5th and 95th percentile (whiskers). * p<.05 for intergroup comparison of septic survivors vs. nonsurvivors. **B.** Time course of LBP plasma concentrations in postoperative septic patients during the first 14 days of sepsis (n = 59). Data are presented as box-plots (open, survivors, n = 40; hatched, nonsurvivors, n = 19) depicting the lower and upper quartiles (boxes) and the 5th and 95th percentile (whiskers). * p<.05 for intergroup comparison of septic survivors vs. nonsurvivors.

Plasma levels of LBP and CRP serially measured at day 1 (onset of sepsis), d2, d3, d5, d7, d10 and d14 are depicted in Figure 2A–B. During the first week of sepsis, both CRP and LBP showed the highest plasma levels at d2 or d3 after onset of sepsis with median values above 100 mg/L and 50 mg/L, respectively, in both groups (S and NS). During the first five days of sepsis, there was no significant difference between S and NS in both plasma markers. Toward the end of the first week, we observed a decline in LBP and CRP to lower levels that was more pronounced in survivors than in nonsurvivors. With a nadir at d5 in nonsurvivors, LBP and CRP levels started to markedly increase within the second week. Surprisingly, we also found moderately increasing LBP and CRP levels from d7 to d14 in the surviving group. In contrast, there was no significant change in white blood count (WBC) during this time period (15363±6860/µl at day 7; 15959±6264/µl at day 10; 11082±3098/µl at day 14; WBC ± SD). The only significant difference between S and NS during the entire study period was observed for CRP at d7 (p = .049).

### Performance of LBP and CRP to predict outcome and correlation of both markers

**Figure 3:**
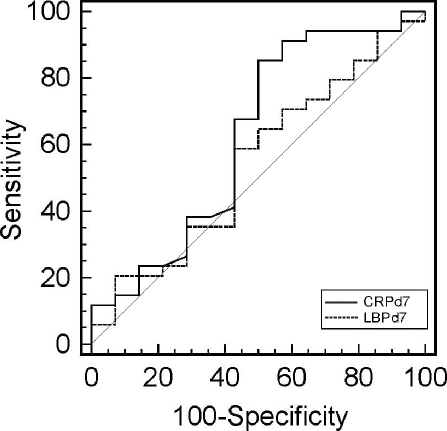
Receiver operating characteristic (ROC) curves of CRP and LBP on day 7 after onset of sepsis analyzed for prediction of survival on day 28. The area under curve (AUC) of CRP and LBP, a measure of predictive accuracy, was 0.63 (CI 0.48–0.76; p = 0.16) and 0.55 (CI 0.40–0.69; p>0.5), respectively.

To evaluate the predictive accuracy of CRP and LBP plasma levels in discriminating survivors from nonsurvivors, ROC analysis was performed. During the first 5 days of sepsis, median plasma concentrations of LBP and CRP were even higher in S than in NS and did not discriminate both groups as assessed by ROC analysis (AUC<.55; data not shown). Only at day 7, CRP levels poorly discriminated S from NS with an area under the curve (AUC) of 0.63 (CI 0.49–0.76; p = 0.16), whereas LBP again failed to discriminate both groups (AUC = 0.55, CI 0.40–0.69; p>0.5) (Figure 3). Pairwise comparison of ROC curves of LBP and CRP showed no significant difference between areas under curve (p = 0.50).

**Figure 4:**
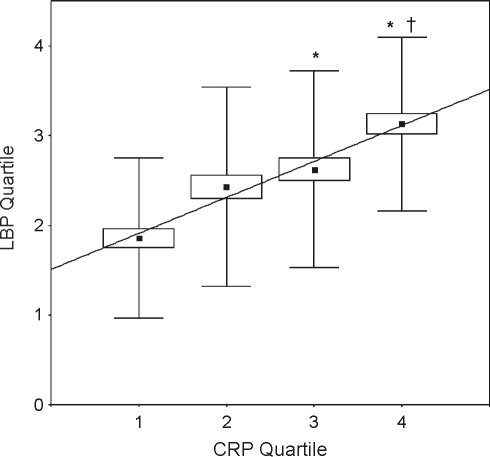
Relationship between CRP and LBP plasma concentrations after transformation to quartiles (Q1–Q4). Data represent the regression of CRP on LBP quartiles and the correlation between CRP and LBP (r^2^ = 0.95, p = 0.16). For every CRP quartile, mean, lower and upper quartile (boxes) and standard deviation (whiskers) of the LBP quartiles are shown. * p<.05 for intergroup comparison of the mean of LBP quartiles associated with Q3 and Q4 vs. Q1 of CRP.

For determination of the relationship between LBP and CRP, plasma levels were transformed to quartile numbers (1–4) according to the quartile (Q1: 0–25th, Q2: 26th–50th, Q3: 51st–75th, Q4: 76th–100th percentile) they were located, in order to account for a variable degree of association between LBP and CRP. Quartile numbers of LBP and CRP plasma concentrations that were obtained from a patient at the same time point were found to highly correlate with each other (r^2^ = .95, p = .02), i.e. the higher the quartile a patient's CRP level was assigned to, the higher the quartile of its concomitant LBP level (Figure 4). Moreover, the mean quartile number of LBP associated with the 3rd and 4th CRP quartile, respectively, was significantly above that of the 1st CRP quartile. In addition, we also studied whether a change (increase or decrease) of CRP from one time point to the next following was associated with a concomitant change of the LBP concentration in the same direction. There was a strong concordance of day-to-day changes of CRP levels (n = 242) with the associated LBP changes for all patients and time points studied during the first 14 days of sepsis (Table 2). In 76% of all cases, where CRP concentrations increased or decreased from one time point to the next, LBP behaved accordingly. Only twelve percent of all CRP increases and 12 percent of all decreases, from one observation day to the next, were not accompanied with a simultaneous change of LBP concentration in the same direction.

**Table 2:** Association of CRP and LBP changes in the plasma concentration from one time point of observation to the other during 14 days after onset of sepsis.

	CRP increasefrom one to the next observation	CRP decreasefrom one to the next observation
**LBP increase**from one to the next observation	**28%**(67/242)	**12%**(29/242)
**LBP decrease**from one to the next observation	**12%**(29/242)	**48%**(117/242)

Data represent the percentage of increases and decreases in CRP levels from one to the next observation that were accompanied by concomitant increases or decreases in LBP levels over all patients and time points measured. Changes of CRP and LBP levels in the same direction (concordance) occurred in 76%, changes in the opposite direction (discordance) in 24% of all cases.

## Discussion

The present study demonstrates that LBP and CRP plasma concentrations have a similar time course during the first 14 days of postoperative sepsis: First an increase with a maximum around the first or second day after onset of sepsis followed by a decrease in the first week and a re-increase in the second week. Plasma concentrations of both LBP and CRP in nonsurvivors did not significantly differ from survivors and were rather lower than those in survivors during the first five days of sepsis. Similar findings have been reported on LBP by other investigators, showing either no difference between S and NS of severe sepsis [15], [24] and critically ill patients [22], respectively, or significantly higher LBP levels in survivors than in nonsurvivors of severe sepsis at study entry [23]. In a mixed study population with SIRS, sepsis and severe sepsis, the maximum LBP concentration during the first 3 days in the ICU was found to moderately discriminate between S and NS [24]. However, as LBP plasma levels have been shown to correlate with sepsis severity [12], [22], differences in LBP plasma concentrations between S and NS are to be expected in inhomogeneous populations with different levels of severity. Therefore, our finding that LBP and CRP concentrations in nonsurvivors increased above that of survivors only from day 7 to day 14, but not in the first three days, may rather be due to the relatively good homogeneity of our study group regarding sepsis severity at study entry.

In the second week of sepsis, we found a marked re-increase of LBP and CRP in the nonsurviving group most likely as part of an inflammatory response to a recurrent or unresolved infection. Surprisingly, we also observed a moderate, but noticeable rise in LBP and CRP plasma levels from day 7 to day 14 in patients of the surviving group without any evidence for a new or recurrent infection. That is, we neither observed clinical and radiological signs nor significant changes in body temperature (data not shown) or white blood count during the second week in group S. We therefore assume that in these patients LBP and CRP unspecifically increased due to stressful maneuvers imposed upon them in the ICU (i.e. end of analgosedation, 9 and extubation, vigorous physiotherapy etc.). Therefore, a moderate re-increase in LBP or CRP plasma concentration in the second week of sepsis is no reliable indicator of a recurrent infection in this setting of postoperative sepsis.

Due to a broad overlap between S and NS both in LBP and CRP plasma concentrations during the first 5 days of sepsis, their predictive accuracy for mortality (at day 28) as assessed by ROC-analysis was very poor (AUC<.55). Similar findings were reported for LBP (AUC = .53) and CRP (AUC = .56) plasma concentrations on ICU admission in a heterogeneous population of postoperative patients with SIRS, sepsis or septic shock [24]. Likewise, Prucha et al [22] found no significant difference in LBP concentrations between survivors and nonsurvivors at study entry in a mixed group of critically-ill patients. In a more recent study on patients with severe sepsis, Villar et al. confirmed this result for LBP at study entry (AUC: 0.57, CI: 0.52–0.71, p = .173). However, 48 hours later, LBP plasma concentration was found a better outcome predictor (AUC: 0.71, CI: 0.61–0.80, p<.0001) [15]. Although the population of that study seems to be quite comparable to our study regarding sepsis severity (mean APACHE II score 23.2 vs. 22.5) and mortality (40% vs. 32%), there may be two relevant differences in the populations that would explain why we could not reproduce Villar's finding that LBP at 48 h is a good predictor of outcome: First, we studied only postoperative septic patients, whereas Villar's study seemed to include medical patients with sepsis and pneumonia as well. As shown for CRP and other acute phase proteins [26], postoperative LBP plasma concentrations could be unspecifically elevated due to surgical trauma. In the present study, therefore, possible differences between S and NS in the LBP levels 48 h after onset of sepsis might have been blurred by an unspecific response to surgery.

Second, in contrast to our study, where only 2 (3%) of the patients had Adult Respiratory Distress Syndrome (ARDS), 55% of Villar's patient population suffered from ARDS [15]. Those ARDS patients as well as the nonsurvivors were reported to have the highest values of LBP, whereas patients who survived and those who did not develop ARDS had significantly lower LBP levels at 48 h [15]. As previously has been shown, LBP can be produced by epithelial cells of the lungs under pathophysiological conditions found in ARDS in response to inflammatory mediators (IL-1, IL-6 and TNF alpha) [16]. Therefore, in Villar's study, the difference in LBP levels between NS and S may rather be attributed to a higher percentage of ARDS patients in the nonsurviving group and not merely to sepsis.

Our finding that CRP plasma levels either did not (d1–d5) or only poorly (AUC = 0.63 at d7) discriminate S from NS during the first week of sepsis, is in good agreement with the studies of Villar, Sakr and other previous investigations [10], [15], [24]. Furthermore, for the entire population studied and across all observation time points, we found a strong correlation between the quartile numbers LBP and CRP plasma concentrations were assigned to (r^2^ = .95, p = .02). This result is not unexpected, since CRP and LBP are both acute phase proteins, induced by the same inflammatory mediators, and correlate significantly well with each other (r = .71, p<.001) in an experimental LPS-inhalation study in healthy volunteers [27]. A strong correlation (r = 0.84, p<.0001) between LBP and CRP was also found by Gaini et al. in patients with suspected community-acquired infection and sepsis [12] and in surgical ICU patients with sepsis syndromes (r^2^ = .36, p<.001) [24]. Likewise, a significant, but less strong correlation (r = .54, p = .002) between LBP and CRP was described in patients with sepsis and septic shock [22].

Although the liver is the main source of both acute phase proteins, under certain pathophysiological conditions, CRP can also be produced by epithelial cells of the kidneys [28] as well as LBP can be released by epithelial cells of the intestine and the lungs [16]. Therefore, the degree of correlation between CRP and LBP may vary, dependent on the individual contribution of extrahepatic sources according to the site of infection.

More often than the absolute plasma concentration of a biomarker, its time course is used to judge the resolution of sepsis. We therefore questioned, whether a change in LBP plasma level from one observation day to the next occurs in parallel with CRP. Our results show that across all time points and all patients studied, more than three out of four changes in the LBP concentration from one to the next observation day were accompanied with a simultaneous change in the CRP level in the same direction. Although LBP is known to significantly increase in plasma approximately 12 hrs ahead of CRP in response to inflammatory stimuli [29], the potential of LBP to get some earlier information regarding the development of sepsis, does not really provide an advantage in a clinical setting where biomarkers are determined in 24-hour intervals. Therefore, our finding does not support the notion that assessment of LBP has significant advantage over CRP in this context. LBP and CRP plasma concentrations, assessed in intervals of 24 hours and more, rather show a very similar kinetics in the course of sepsis. Given the lower costs and the long-term clinical experience with CRP measurements, we do not consider LBP as a useful biomarker for the monitoring of sepsis in patients post surgery.

Our study has several strengths. First, according to Randolph et al. [30], our study population was a representative sample of critically ill adult patients and sufficiently homogenous with respect to prognostic outcome, since all patients had sepsis after major surgery and were studied from day 1 of sepsis. Second, the unbiased and well-defined endpoint used was mortality directly related to multiple organ dysfunction due to severe sepsis within the first 28 days.

However, some limitations also merit consideration: The prognostic accuracy of LBP and CRP may have been negatively influenced in our study population, because LBP and CRP are frequently unspecifically elevated after major surgery to levels comparable to those seen in sepsis. Therefore, in septic patients not subjected to surgical trauma, e.g. in medical patients, LBP and CRP may provide a better prognostic accuracy. In addition, although statistically controlled, we cannot completely exclude small sample size effects in our study population. Moreover, LBP and CRP may be clinically useful in critically ill neonates and children, where LBP has a high diagnostic accuracy to differentiate sepsis from non-infectious SIRS [14], [31].

In conclusion, our study demonstrates that LBP and CRP plasma concentrations are well correlated with each other and change concordantly in the course of sepsis. Furthermore, LBP and CRP plasma levels have quite the same predictive value and are neither suitable to monitor resolution of sepsis nor sufficiently reliable to detect a recurrent infection during the first 14 days of sepsis in adult postoperative patients.
